# Slumber’s Duel: The Upstart Orexin Receptor Antagonists Versus the Battle-Hardened Z-Drugs: Systematic Review and Meta-Analysis Unveiling a Tale of Efficacy and Safety of Two Contenders

**DOI:** 10.1192/bjo.2025.10086

**Published:** 2025-06-20

**Authors:** Asha Devi Dhandapani, Sathyan Soundara Rajan, Gaurav Uppal, Sneh Babhulkar, Betsy Marina Babu

**Affiliations:** ^1^BCUHB, Wrexham, United Kingdom; ^2^Satyam Hospital, Ludhiana, India; ^3^Greater Glasgow and Clyde NHS Trust, Glasgow, United Kingdom; ^4^London and KSS school of Psychiatry, London, United Kingdom

## Abstract

**Aims:** Primary insomnia, a separate diagnosis that is now included within the newly broader categorization of insomnia, greatly affects the quality of life. This meta-analysis evaluates the efficacy and safety of orexin receptor antagonists (ORAs) and Z-drugs for insomnia in adults.

Pharmacological approaches to the management of insomnia include the use of our own rendition of those generic drugs commonly referred to as ORAs and Z-drugs. Z-drugs are mainly used; nevertheless, doubts as to their long-term security remain. Targeted at orexin receptors, ORAs are novel. This system consolidates knowledge for use in clinical evaluation and management.

**Methods:** Accordingly, a Cochrane-Central Register of Controlled Trials Database, a Systematic review using the keywords, ORAs, and Z-drugs was conducted. The criteria for patient inclusion involved all adults diagnosed with insomnia. Measurements of the extent of benefits from the interventions were: Total sleep time, sleep onset latency, and adverse effects.

Bias was determined using SRR and overall risk of bias was determined using the ROB 2 tool. This meta-analysis was conducted by applying random effects models.

**Results:** Six trials showed that ORAs shortened sleep onset latency compared with zolpidem and other Z-drugs (mean difference −15.3 min, 95% CI −22.1 to −8.5). Total sleep time was similar to total time between sleep onset and wake-up in both groups. ORAs demonstrated a superior safety profile, with lower incidence of next-day somnolence (risk ratio: 0).

This was associated with a decreased risk for cognitive impairment at follow up (risk ratio: 0.65, 95% CI: 0.52–0.81) and for dependency (risk ratio: 0.38, 95% CI: 0.25–0.58).

According to the funnel plot analysis there was no significant publication bias that exists within the studies.

**Conclusion:** They [ORAs] are at least as effective as the Z-drugs in the management of insomnia and are safer in terms of next-day implications and withdrawal especially in elderly patients. These experiments affirm using ORAs as a first-line pharmacological remedy in chronic insomnia in adults.

